# CT Features of Granulomatous–Lymphocytic Interstitial Lung Disease (GLILD): The “Kebab Sign” as a Marker to Support Differential Diagnosis

**DOI:** 10.3390/diagnostics16101496

**Published:** 2026-05-14

**Authors:** Federica Ciccarese, Nicolò Piva, Marco Carpano, Ilaria Bassi, Aldo Guerrieri, Gioacchino Schifino, Stefano Nava, Cristina Mosconi, Maurizio Zompatori

**Affiliations:** 1 Department of Radiology, IRCCS Azienda Ospedaliero-Universitaria di Bologna, 40138 Bologna, Italy; cristina.mosconi@aosp.bo.it; 2 Respiratory and Critical Care Unit, IRCCS Azienda Ospedaliero-Universitaria di Bologna, 40138 Bologna, Italy; marco.carpano@aosp.bo.it (M.C.); ilaria.bassi@aosp.bo.it (I.B.); aldo.guerrieri@aosp.bo.it (A.G.); stefano.nava@unibo.it (S.N.); 3 Respiratory Medicine Unit, Department od Cardio-Thoracic-Vascular Pathologies, Arcispedale Sant’Anna, Via A. Moro 8, 44124 Ferrara, Italy; gioacchino.schifino@ospfe.it; 4 Alma Mater Studiorum, Department of Medical and Surgical Sciences (DIMEC), University of Bologna, 40138 Bologna, Italy; maurizio.zompatori@unibo.it

**Keywords:** CVID, GLILD, Kebab sign, sarcoidosis, granulomatous lung disease, air bronchogram, BID, DLD

## Abstract

**Objective:** In this study, we aimed to evaluate high-resolution computed tomography (HRCT) features of granulomatous–lymphocytic interstitial lung disease (GLILD) in patients with Common Variable Immunodeficiency (CVID), and to describe a novel imaging feature—termed the “Kebab sign”—as a potential radiologic marker of GLILD. **Materials and Methods:** We retrospectively reviewed HRCT scans of 15 patients with GLILD diagnosed between 2005 and 2025 at a single institution (seven biopsy-confirmed, eight probable diagnoses based on multidisciplinary consensus). CT patterns were assessed for predominant morphology (nodular, reticular, alveolar, fibrotic), distribution (axial and cranio-caudal), and presence of extra-parenchymal findings. Nodules were characterized by size, density, morphology, and the presence of air bronchograms. The “Kebab sign” was defined as nodules aligned along bronchial structures with associated peribronchial thickening. **Results:** All patients demonstrated a diffuse nodular pattern, with non-calcified macronodules in 100% and micronodules in 60% of cases. Air bronchograms were present in 87% of macronodules. A peri-bronchovascular distribution with lower lung predominance was observed in the majority of cases. The “Kebab sign” was identified in 87% of patients. Splenomegaly and hilar/mediastinal lymphadenopathy were observed in 75%. In 20% of patients, fibrosing features were also present, particularly in older individuals. **Conclusions:** HRCT findings of GLILD typically include peri-bronchovascular nodules with lower lobe predominance, typically associated with splenomegaly and mediastinal lymphadenopathy. The newly described “Kebab sign,” reflecting nodular alignment along thickened bronchial structures, may represent a useful imaging clue to support the diagnosis of GLILD.

## 1. Introduction

Common Variable Immunodeficiency (CVID) encompasses a heterogeneous group of primary immunodeficiency disorders characterized by hypogammaglobulinemia and impaired antibody responses, resulting in suboptimal responses to vaccination and an increased susceptibility to recurrent respiratory infections.

CVID is the most frequent symptomatic primary immunodeficiency, although it remains a rare condition overall. The estimated prevalence ranges from approximately 1 in 25,000 to 1 in 50,000 individuals in European and North American populations, with a balanced distribution between males and females and no evident ethnic predilection. Diagnosis can occur at any age, but most cases are identified after puberty, typically between 20 and 45 years of age. Significant diagnostic delays are common, with a median time to diagnosis of around 4–5 years from the onset of initial symptoms, reflecting the clinical complexity and phenotypic variability of the disease. Pulmonary involvement may include benign lymphoproliferative disorders, airway disease, interstitial lung disease (ILD), and primary and secondary neoplasms [1]. Although ILDs have been described in association with CVID, the most frequent and characteristic pulmonary manifestation is granulomatous–lymphocytic interstitial lung disease (GLILD), occurring in an estimated 10–25% of patients with CVID. The presence of GLILD is associated with increased morbidity and negatively influences clinical prognosis if not promptly identified and appropriately managed [2,3,4].

GLILD is defined, according to the British Lung Foundation/United Kingdom Primary Immunodeficiency Network, as a distinct clinicopathological entity occurring in patients with CVID, characterized by a combination of granulomatous inflammation and lymphoid infiltration of the lung parenchyma, in the absence of an identifiable infectious or malignant cause [5]. In the same consensus, no specific radiological features were deemed mandatory for the diagnosis of GLILD.

However, the diagnosis of GLILD currently requires a compatible radiological pattern in conjunction with histopathological confirmation via lung biopsy. The presence of characteristic features on high-resolution computed tomography (HRCT) in the absence of biopsy may support a presumptive diagnosis.

There is broad consensus in the literature regarding certain characteristic features of GLILD, namely a bilateral nodular pattern with predominant basal distribution, in association with hilar and mediastinal lymphadenopathies and splenomegaly.

Galant-Swafford et al. [1] recently proposed a classification of HRCT patterns into three categories: typical of GLILD, compatible with GLILD, and suggestive of alternative diagnoses.

In both typical and compatible patterns, hilar and mediastinal lymphadenopathy and splenomegaly are present, thus representing two key elements for radiological diagnosis. The typical pattern also shows the characteristic distribution (peri-bronchovascular and lower zone predominance) and is characterized by the coexistence of pulmonary nodules, ground-glass opacities and consolidations. By contrast, in compatible pattern, radiological presentation could vary in distribution and findings.

Patients with a diagnosis of CVID, particularly those with pulmonary involvement, routinely undergo periodic follow-up with high-resolution computed tomography (HRCT). In addition, HRCT may be performed to evaluate the onset or worsening of respiratory symptoms. In both scenarios, it is crucial to distinguish active disease from superimposed inflammatory or infectious processes. The identification of pulmonary involvement with imaging features that are not clearly attributable to either condition complicates clinical management. In particular, the presence of pulmonary infiltrates that fail to respond to standard antibiotic therapy or show delayed resolution often necessitates further diagnostic evaluation, including lung biopsy.

The aim of the present study was to analyze CT features of patients with GLILD, to confirm and assess radiological criteria for diagnosis.

Secondly, we firstly described the association of nodular components and peri-bronchovascular involvement (typical features in GLILD) as the “Kebab sign”.

## 2. Materials and Methods

Fifteen cases of GLILD were identified at our institution between 2005 and 2025 (7 cases with a biopsy-confirmed diagnosis and 8 cases with a probable diagnosis determined in multidisciplinary meeting based on clinical, laboratory, and imaging findings).

The study included HRCT scans performed at disease onset or the earliest available imaging showing radiologically visible lung disease. Examinations were performed using multidetector CT scanners according to standard clinical protocols for interstitial lung disease evaluation. The scanner was a 128-slice CT scanner (Ingenuity Core 128; Philips Healthcare, Cleveland, OH, USA), with the following technical parameters: tube voltage, 120 kV; tube current modulation, 100–250 mAs; spiral pitch factor, 1.224. Scans were acquired in the supine position at full inspiration, from the lung apices to the costophrenic angles. Images were obtained using thin-section acquisition with a collimation of approximately 0.6–1.25 mm and reconstructed with a slice thickness of 0.6–1.25 mm and an increment of 0.5–1.0 mm. Reconstructions were generated using a high-spatial-frequency (sharp) algorithm optimized for lung parenchyma evaluation. In addition to standard axial images, multiplanar reconstructions (axial, coronal, and sagittal planes) were routinely available and used for image interpretation. Maximum intensity projection (MIP) and minimum intensity projection (MinIP) reconstructions were also applied to enhance the visualization of nodular structures, bronchovascular anatomy, and areas of decreased attenuation. Images were reviewed using standard lung window settings (window width 1500–1600 HU; window level −500 to −600 HU) and mediastinal window settings (window width 350–400 HU; window level 40–60 HU). Intravenous contrast medium was not routinely administered and was used only when clinically indicated to assess mediastinal structures or vascular abnormalities.

Features of GLILD were evaluated in terms of the predominant disease pattern (nodular, reticular, alveolar, fibrotic), axial gradient (central vs. peripheral, with particular attention to the predominant type of interstitial involvement—peri-bronchovascular and interlobular), and cranio-caudal gradient (upper, middle, and lower lung zones, defined respectively by the planes passing through the inferior margin of the aortic arch and the inferior margin of the right pulmonary vein).

Non-calcified nodules were classified as macronodules (>6 mm) and micronodules (<6 mm), and further categorized as solid, subsolid, or nodules with a halo sign. For macronodules, the presence of air bronchograms was assessed.

The extra-parenchymal features considered included mediastinal lymphadenopathy and splenomegaly (as permitted by the extent of the acquisition volume).

HRCT images were independently reviewed by two experienced thoracic radiologists. In cases of discrepancy, consensus was reached through direct discussion between the observers. All radiological features, including the presence of the “Kebab sign,” nodule distribution, and peri-bronchovascular involvement, were recorded according to predefined criteria to ensure consistency and reproducibility of the analysis.

## 3. Results

All 15 patients included in the study (12 females, 3 males; mean age 40 years [IQR 32–48 years]) presented with diffuse lung disease characterized by a nodular pattern. Imaging findings are summarized in Table 1.

### 3.1. Nodules

Specifically, all cases showed the presence of non-calcified macronodules with variable profusion, associated with micronodules in 60% of cases. The nodules most frequently appeared solid; in some cases, subsolid nodules (30%) and/or nodules with a halo sign (13%) were also observed. Notably, in patients for whom serial imaging was available, the nodules varied over time in both number and morphology. We highlight that in 87% of cases, at least some of the macronodules demonstrated an air bronchogram.

**Figure 1:**
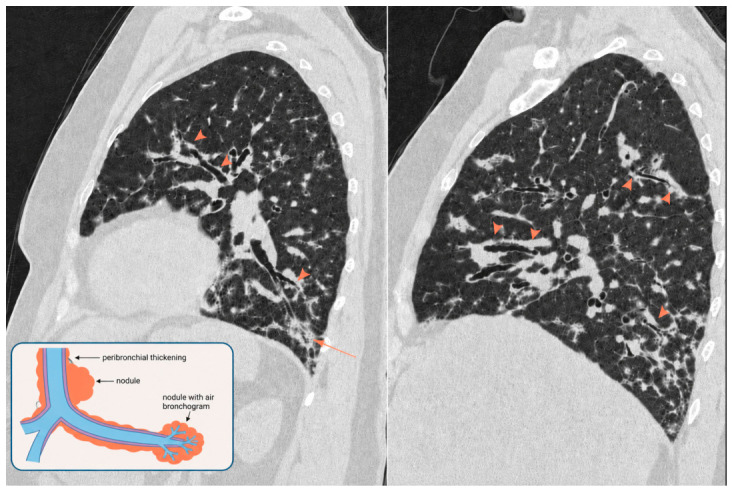
Sagittal non-contrast CT images show multifocal irregular (cylindric and nodular) peri-bronchial thickening, the Kebab sign (arrow head); the sign is better appreciated and should be sought on the sagittal plane; on the right, in the posterior costophrenic angle, a bronchocentric nodular consolidation is visible (arrow).

The nodules are typically aligned along bronchial ramifications and often appear to be in continuity with the aforementioned thickening, especially in the peripheral lung. We describe these combines features as the “Kebab sign”. This distinctive feature was present in 87% of our cases (Figure 1).

### 3.2. Patterns

**Figure 2:**
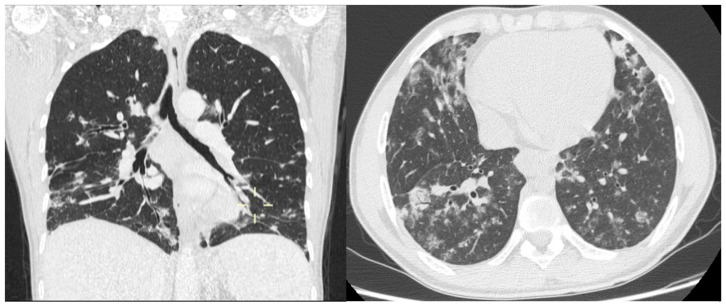
Left image: the findings described as the “Kebab sign” are predominantly located in the lower lung zones, with relative sparing of the upper lobes. Right image: involvement of the lower lung zones with a large solid peripherally located (mantellar) nodule exhibiting an air bronchogram.

In terms of distribution pattern, GLILD predominantly affects the lower pulmonary regions. Involvement of the upper lobes may be observed, but typically only in the setting of widespread disease involving the entire lung parenchyma (Figure 2).

The nodular pattern showed a clear peri-bronchovascular distribution, manifesting as broncho-centric macronodules and micronodules, or as macronodules with air bronchograms. Involvement of the perilobular interstitium was observed in only 20% of cases, and always in association with prominent involvement of the central (peri-bronchovascular) interstitium.

We emphasize the nodular component of the disease is almost constantly associated with another feature that, in our opinion, has not been sufficiently addressed in the literature, namely, the presence in GLILD of a multifocal/diffuse, confluent peri-bronchial and peri-bronchiolar thickening, which is consistent with involvement of the bronchus-associated lymphoid tissue (BALT). Indeed, CVID could also determine airway disease.

### 3.3. Bronchiectasis

According to our findings, bronchial dilation was observed in only a small proportion of cases (15–20%) and does not represent a distinctive feature of GLILD. It should be noted that peri-bronchial thickening and nodules with an air bronchogram create a visual contrast between the dark lumen of the bronchi and the surrounding dense parenchymal opacities. This contrast may exaggerate the apparent bronchial caliber on HRCT images, potentially leading to overestimation of bronchial dilatation. Furthermore, bronchiectasis is defined as a clinical entity characterized by irreversible bronchial dilatation [6], whereas the bronchial abnormalities observed in association with GLILD-related consolidations regress following resolution of the parenchymal opacities. An exception is represented by traction bronchiectasis, which may be observed in cases demonstrating fibrotic progression.

### 3.4. Extra-Parenchymal Findings

**Figure 3:**
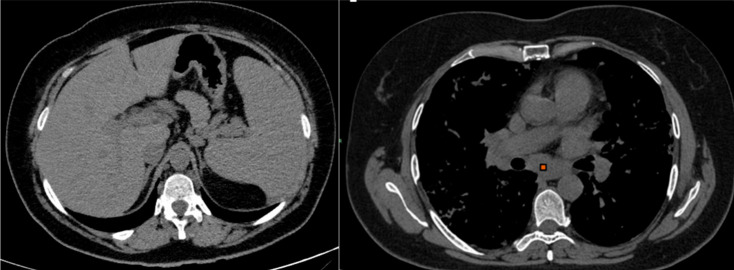
Axial non-contrast CT images show splenomegaly and enlarged subcarinal lymph nodes (red square). The coexistence of splenomegaly, enlarged mediastinal lymph nodes, and interstitial lung disease with a nodular pattern should raise suspicion for an underlying lymphoproliferative disorder.

Moreover, our case series demonstrated that in approximately 75% of cases, GLILD was associated with extensive hilar and mediastinal lymphadenopathy, along with marked splenomegaly. Lymph node enlargement was typically bilateral and symmetric, without necrotic feature (Figure 3).

### 3.5. Lung Fibrosis

In three patients from our case series, the earliest available HRCT revealed, in association with the characteristic nodular pattern previously described, a diffuse fibrosing lung disease predominantly affecting the lower lobes. The fibrotic pattern was characterized by reticular abnormalities, architectural distortion, and, in some cases, early traction bronchiectasis. Honeycombing was not a predominant finding. The association between older age and fibrotic manifestations in our cohort may suggest that fibrosis represents a late-stage or progressive phenotype of GLILD.

### 3.6. Pulmonary Function Test

In the group with the Kebab sign, the mean values were as follows: FVC 3.33 ± 0.96 L, FVC 96.8 ± 19.0% of predicted, FEV1 2.79 ± 1.08 L, FEV1 93.6 ± 22.1% of predicted, and FEV1/FVC ratio 62.3 ± 17.4%. In the group without the Kebab sign, the corresponding mean values were FVC 2.69 ± 0.31 L, FVC 99.0 ± 8.5% of predicted, FEV1 2.04 ± 0.28 L, FEV1 89.5 ± 9.2% of predicted, and FEV1/FVC ratio 67.5 ± 3.5%. Data are summarized in Table 2.

Exploratory comparisons between the two groups did not reveal any statistically significant differences for any of the parameters analyzed: FVC in liters (*p* = 0.269), FVC % of predicted (*p* = 1.000), FEV1 in liters (*p* = 0.229), FEV1% of predicted (*p* = 0.800), and FEV1/FVC ratio (*p* = 0.798).

Overall, the data do not demonstrate a statistically significant association between the presence of the Kebab sign and impairment of respiratory functional parameters.

It should be noted that, as the aim of the study was to identify features useful for the diagnosis of GLILD, a quantitative assessment of the extent of the Kebab sign was not performed, although such an evaluation might correlate with the presence of obstructive impairment.

## 4. Discussion

The diagnosis of GLILD is, in practice, a diagnosis of exclusion, given the wide range of conditions included in its differential diagnosis [7]. In particular, the differential diagnosis includes, as previously mentioned, pulmonary infections, other forms of interstitial lung disease, lymphoproliferative disorders and other granulomatous conditions.

### 4.1. Infections

The appearance of isolated consolidations and/or ground-glass opacities in a patient with CVID should primarily raise suspicion of an infectious etiology. Even their presence in association with a nodular pattern compatible with GLILD is suggestive of an inflammatory overlap and requires appropriate clinical–laboratory assessment and therapeutic management. Persistent consolidations and/or ground-glass opacities may reflect organizing phenomena or be a manifestation of diffuse lung disease [8].

**Figure 4:**
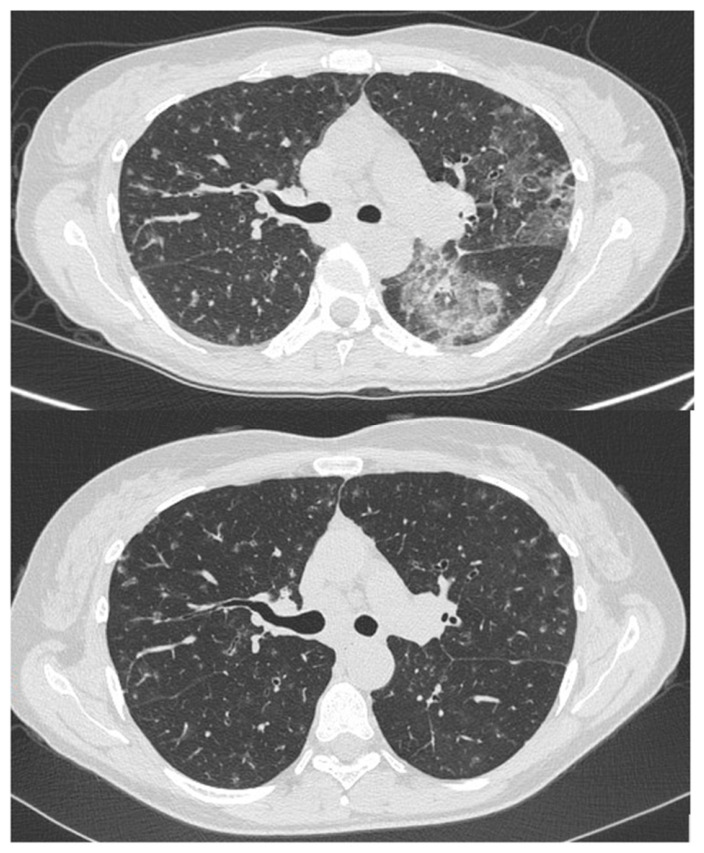
Upper image: extensive ground-glass opacities involving the apical segment of the left lower lobe and the lingula in a patient with a diagnosis of GLILD. Lower image: follow-up scan after 3 months shows complete resolution of the previously described ground-glass opacity which has been demonstrated to represent a superimposed infection due to *Haemophilus influenzae.* Multiple solid and subsolid bronchiolocentric nodules and micronodules persist in both lungs, consistent with the underlying disease.

In our opinion, the presence of consolidations and GGO, although relatively frequent, should not be included in the definition of a characteristic radiological pattern of GLILD. This is because these findings have a broad differential diagnosis and cannot be reliably attributed to a granulomatous or lymphocytic origin (Figure 4). Furthermore, based on our experience, only the presence of a nodular pattern with the specific characteristics illustrated, in association with splenomegaly, allows the consolidations/ground-glass opacities to be attributed to GLILD, provided the clinical and laboratory context is consistent.

A bilateral and symmetrical nodular pattern is an uncommon manifestation of infectious diseases; however, it should be considered and ruled out in particular in immunocompromised patients. It is noteworthy that the nodules observed in GLILD do not exhibit cavitation [9,10].

### 4.2. Diffuse Lung Disease

Diffuse lung diseases that consistently overlap with GLILD in the differential diagnosis are organizing pneumonia (CVID-OP) and lymphocytic interstitial pneumonia (LIP).

Organizing pneumonia (OP) is characterized by patchy consolidations and ground-glass opacities with peri-bronchovascular and/or peripherical distribution and lower lobe predominance. Multiple ill-defined nodules may occur as isolated findings or, more commonly, in association with consolidations. These imaging features closely resemble those observed in GLILD. In particular, the nodules in OP typically have ill-defined margins, may demonstrate the halo sign, show peri-bronchovascular distribution, and can exhibit air bronchograms within the lesions. However, OP usually lacks the typical diffuse peri-bronchial thickening described in GLILD, and the nodular pattern is generally not predominant. Moreover, the atoll sign (reversed halo) and perilobular opacities (perilobular sign), which can be seen in OP, have not been reported in GLILD [11,12,13].

Lymphocytic interstitial pneumonia (LIP) shares several features with GLILD, including patchy consolidations or ground-glass opacities and involvement of the peri-bronchovascular lymphatics, manifesting as peri-bronchial thickening and ill-defined centrilobular nodules. However, GLILD typically lacks involvement of the perilobular lymphatics, which in LIP appears as nodular thickening along the interlobular septa. Moreover, the presence of significant cystic changes, which is a hallmark finding in LIP, has not been described in GLILD. Both LIP and OP may present with hilar and mediastinal lymphadenopathy, whereas splenomegaly is not typically associated with either condition [14,15].

Finally, GLILD may progress to a fibrosing phenotype, with the most frequent patterns being non-specific interstitial pneumonia (NSIP) and indeterminate for usual interstitial pneumonia (UIP). Similar to earlier stages, these fibrosing changes predominantly affect the lower lung zones. There may also be coexistence of a nodular pattern, indicative of active disease, alongside the fibrosing pattern.

### 4.3. Non-Infectious Granulomatous Diseases

The differential diagnosis of non-infectious granulomatous lung diseases is broad and beyond the scope of this work. Instead, the focus here is on the differential diagnosis with sarcoidosis.

Pulmonary sarcoidosis exhibits a wide range of radiological presentations. In some cases, the imaging features are distinctive and do not pose diagnostic challenges in differentiating from GLILD, for example, micronodules “avid of pleura” with high profusion along interlobular septa, consolidations appearing as a “galaxy sign”, ground-glass opacities appearing as a “nodular cluster sign”, and subpleural pseudo-plaques. However, the differential diagnosis becomes more challenging when sarcoidosis presents almost exclusively with aspecific consolidations and ground-glass opacities or with a nodular pattern with peri-bronchovascular distribution (“nummular” sarcoidosis), which can closely mimic GLILD. In these cases, certain features may help guide the diagnosis: GLILD predominantly affects the lower lung zones, whereas sarcoidosis typically involves the upper lung zones. Nodules in GLILD very often show an air bronchogram, a finding that is relatively uncommon in sarcoidosis [16]. In nummular sarcoidosis, clusters of nodules are often visible, while GLILD has a diffuse homogenous or sporadic distribution [17,18,19].

Hilar and mediastinal lymphadenopathy is a common finding in both conditions. However, the presence of lymph node calcifications is not typical of GLILD and should prompt consideration of an alternative diagnosis. On the other hand, although splenomegaly can occur in sarcoidosis, its presence in patients with CVID, in conjunction with the aforementioned parenchymal pattern, strongly supports a diagnosis of GLILD [20].

Eosinophilic granulomatosis with polyangiitis may present with centrilobular nodules and peri-bronchial thickening; however, mediastinal lymph node involvement is rare. In granulomatosis with polyangiitis, a nodular pattern, even when associated with peri-bronchial thickening, is more frequently observed; however, nodules tend to cavitate, and mediastinal lymph node involvement is rare [21,22]. Another rare granulomatous disease with a lower zone predominance and peri-bronchial distribution is lymphomatoid granulomatosis. However, nodules are characterized by angioinvasive features and a tendency to cavitate; moreover, lymph node involvement is uncommon [23,24].

Pulmonary involvement in inflammatory bowel disease presenting as necrobiotic nodules is rare (<1% of cases); however, it should be considered, as it may have an age of onset similar to that of GLILD. Necrobiotic nodules typically show a peripheral and subpleural distribution and tend to cavitate; moreover, concomitant intestinal involvement supports the diagnosis [25,26].

A condition that may radiologically enter the differential diagnosis with GLILD is the so-called sarcoid-like reaction. It is characterized by pulmonary nodules with a peri-lymphatic distribution and significant mediastinal lymph node involvement. In this setting as well, clinical history is crucial: the presence of recognized triggering factors, particularly treatment with immune checkpoint inhibitors, supports the differential diagnosis [27,28].

### 4.4. Lymphoproliferative Disorders

Differentiating GLILD from lymphoproliferative disorders based on imaging alone is challenging, due to overlapping radiological findings and the potential for GLILD to undergo lymphomatous transformation.

Certain benign or indolent lymphoproliferative disorders share features that can mimic GLILD. In particular, focal (nodular) lymphoid hyperplasia and MALT lymphoma often present with solitary, but occasionally multiple, consolidations or nodules, typically exhibiting an air bronchogram. In these cases, the presence of ilo-mediastinal lymphadenopathy and splenomegaly is not as constant as in GLILD. Specifically, MALT lymphoma is the most common form of primary pulmonary lymphoma and, by definition, does not present with extra-thoracic manifestations (e.g., splenomegaly) for at least three months following diagnosis. In general, malignant lymphomas present with lymph node involvement, which may be associated with or without parenchymal involvement. The latter typically manifests as solitary or multiple nodules, consolidations, and thickening of the peri-bronchovascular interstitium—features that are entirely overlapping with those observed in GLILD. It should be emphasized that in GLILD both lymph nodes and parenchymal lesions frequently demonstrate uptake on PET imaging. Consequently, patients with GLILD should undergo follow-up with HRCT, and progressive increase in nodule/mass size despite treatment is the primary indicator raising suspicion for malignant progression [28,29,30].

## 5. Conclusions

The results of our study confirm that diagnosis should be suspected in case of a nodular pattern, with perivascular distribution and lower field predominance, associated with splenomegaly and mediastinal adenopathies. Dimension size of nodules could be variable, as well as density.

Multiplanar reconstructions help in the detection of another distinctive element of GLILD that could help in the diagnosis, i.e., the Kebab sign. This sign reflects the airway disease and the parenchymal involvement, resulting in peri-bronchial thickening and nodules along bronchial ramifications with air bronchogram. It should be carefully taken into consideration and suggest diagnosis when present, especially if a typical pattern could not be addressed (for example in patients with a compatible pattern).

The Kebab sign is not pathognomonic for GLILD, as it may overlap with atypical presentations of sarcoidosis and other rare granulomatous diseases, and it does not allow definitive exclusion of lymphoproliferative disorders. However, its identification may be clinically useful in several respects. First, it can help orient the initial diagnostic workup by raising a radiological suspicion of GLILD and thereby prompting evaluation of the patient’s immunological status. Second, within an appropriate clinical context—particularly in patients with a known diagnosis of CVID—the presence of the Kebab sign may aid in excluding superimposed inflammatory or infectious processes.

The most recent revision of the classification of interstitial pneumonias [31] has highlighted the entity of bronchocentric interstitial pneumonia (BIP), designating it as an overarching category that encompasses distinct radiological and histopathological patterns characterized by predominant broncho-centric involvement. Among the potential etiologies proposed are hypersensitivity pneumonitis (HP), connective tissue disease-associated interstitial lung disease (CTD-ILD), aspiration, and drug-related inhalational or exposure injury. In our opinion GLILD exhibits a comparable distribution pattern, and is most appropriately aligned with the subgroup of non-fibrotic (cellular) BIP. This reconceptualization offers a valuable framework for the differential diagnosis, particularly in distinguishing GL-ILD from sarcoidosis.

The present study has several limitations. First, the relatively small sample size and the retrospective, single-center design may limit the generalizability of the findings. In addition, a formal assessment of inter-observer reproducibility of the described radiological sign was not performed, an aspect that warrants further investigation in future studies. Another limitation is the absence of a control group consisting of patients with granulomatous or lymphoproliferative diseases, which would have allowed for direct comparison and a more accurate evaluation of the specificity of the “Kebab sign”.

Furthermore, the limited number of patients with histological confirmation represents an additional constraint, potentially affecting the diagnostic robustness of the observations. The lack of a quantitative stratification of the extent of the “Kebab sign” may also have limited the ability to detect possible correlations with functional impairment, such as obstructive ventilatory defects. Finally, the absence of expiratory CT acquisitions and contrast-enhanced imaging precluded a more comprehensive evaluation of associated features, including potential air trapping and the assessment of relationships with adjacent vascular structures.

Larger, multicenter studies with standardized imaging protocols, quantitative assessment of radiological findings, and broader histopathological validation are needed to confirm and expand upon these preliminary results, as well as to better define their role in the differential diagnosis, particularly with conditions such as sarcoidosis.

## Figures and Tables

**Table 1:** Imaging findings.

**Distribution**	
*Peribroncovascular only*	12 (80%)
*Periferical only*	0 (0%)
*Both*	3 (20%)
**Predominance**	
*Upper field*	0 (0%)
*Lower field*	15 (100%)
Macronodules	15 (100%)
Micronodules	9 (60%)
**Interlobular thickening**	
*Non-fibrotic*	0 (0%)
*Fibrotic*	3 (20%)
**Bronchiectasis**	
*Non-traction*	3 (20%)
*Traction*	2 (13%)
Kebab sign	13 (87%)
Lymphadenomegaly	11 (73%)
Splenomegaly	11 (73%)
Ground-glass opacites	6 (40%)
Consolidation	4 (27%)

**Table 2:** Pulmonary Function Test.

Tiffenau Index (IT)	FVC (L)	FVC (%)	FEV 1 (L)	FEV1 (%)	DLCO %	Kebab Sign
89	3.21	104.00	2.88	108.00	88.00	Yes
71	2.28	75.00	1.63	62.00	54.00	Yes
82.9	3.90	73.00	3.24	72.00	69.00	Yes
87	4.26	79.00	3.69	82.00	76.00	Yes
77	3.31	102.00	2.57	93.00	74.00	Yes
77	3.06	109.00	2.37	100.00	69.00	Yes
84	2.75	104.00	2.30	103.00	36.00	Yes
77	2.91	105.00	2.24	96.00	65.00	No
75	1.85	68.00	1.39	60.00	35.00	Yes
87	4.01	115.00	3.49	116.00	62.00	Yes
80	3.44	126.00	2.75	118.00	85.00	Yes
91	2.75	87.00	2.52	92.00	46.00	Yes
74	2.47	93.00	1.84	83.00	70.00	No
96	5.59	109.00	5.37	123.00	69.00	Yes
69.6	2.94	108.00	2.05	88.00	47.00	Yes

## Data Availability

The raw data supporting the conclusions of this article will be made available by the authors on request.

## Abbreviations

BALT	Bronchus-Associated Lymphoid Tissue
BIP	Bronchocentric Interstitial Pneumonia
CT	Computed Tomography
CTD-ILD	Connective Tissue Disease-Associated Interstitial Lung Disease
CVID	Common Variable Immunodeficiency
DLD	Diffuse Lung Disease
GGOs	Ground-Glass Opacities
GLILD	Granulomatous–Lymphocytic Interstitial Lung Disease
HP	Hypersensitivity Pneumonitis
HRCT	High-Resolution Computed Tomography
IBD	Inflammatory Bowel Disease
ILD	Interstitial Lung Disease
LIP	Lymphocytic Interstitial Pneumonia
MALT	Mucosa-Associated Lymphoid Tissue
NSIP	Non-Specific Interstitial Pneumonia
OP	Organizing Pneumonia
PET	Positron Emission Tomography
UIP	Usual Interstitial Pneumonia
